# Investigation of Vortex Clouds and Droplet Sizes in Heated Water Spray Patterns Generated by Axisymmetric Full Cone Nozzles

**DOI:** 10.1155/2013/796081

**Published:** 2013-11-05

**Authors:** M. Y. Naz, S. A. Sulaiman, B. Ariwahjoedi, Ku Zilati Ku Shaari

**Affiliations:** ^1^Department of Fundamental and Applied Sciences, Universiti Teknologi PETRONAS, Bandar Seri Iskandar, 31750 Tronoh, Perak, Malaysia; ^2^Department of Mechanical Engineering, Universiti Teknologi PETRONAS, Bandar Seri Iskandar, 31750 Tronoh, Perak, Malaysia; ^3^Department of Chemical Engineering, Universiti Teknologi PETRONAS, Bandar Seri Iskandar, 31750 Tronoh, Perak, Malaysia

## Abstract

The hot water sprays are an important part of many industrial processes, where the detailed knowledge of physical phenomena involved in jet transportation, interaction, secondary breakup, evaporation, and coalescence of droplets is important to reach more efficient processes. The objective of the work was to study the water spray jet breakup dynamics, vortex cloud formation, and droplet size distribution under varying temperature and load pressure. Using a high speed camera, the spray patterns generated by axisymmetric full cone nozzles were visualized as a function water temperature and load pressure. The image analysis confirmed that the spray cone angle and width do not vary significantly with increasing Reynolds and Weber numbers at early injection phases leading to increased macroscopic spray propagation. The formation and decay of semitorus like vortex clouds were also noticed in spray structures generated at near water boiling point temperature. For the nozzle with smallest orifice diameter (1.19 mm), these vortex clouds were very clear at 90°C heating temperature and 1 bar water load pressure. In addition, the sauter mean diameter (SMD) of the spray droplets was also measured by using Phase Doppler Anemometry (PDA) at different locations downstream of the nozzle exit. It was noticed that SMD varies slightly w.r.t. position when measured at room temperature whereas at higher temperature values, it became almost constant at distance of 55 mm downstream of the nozzle exit.

## 1. Introduction

Liquid sprays and corresponding atomizing systems have many industrial applications. Delightful accounts of the earlier research work on liquid jet breakup phenomena and applications may be obtained from the comprehensive review of the literature [1–3]. Whiles a seminal literature has been seen so far on the flow behavior and jet disintegration of heated liquids from axisymmetric swirl type spray nozzles, the process of spraying the hot liquids is one in which the spray jet is disintegrated by thermal and kinetic energies of partially evaporated liquids. The insertion of thermal energy into the spraying system stimulates the partial evaporation of the target liquid. When the liquid is discharged into the surrounding environment, a phase inversion takes place due to spray disintegration, and the vapor phase inside the nozzle raises the level of disintegration. In comparison with highly pressurized atomization, droplet size distributions shift to smaller diameters in thermally energized atomization even at very low load pressures. It combines the benefits of pressure atomization and multiphase atomization and makes the jet disintegration possible even at moderate load pressure [2]. 

Number of key parameters are commonly used to explain the spray jet dynamics in a particular spray processing application. These are the spray angle, spray width, jet breakup length, spray tip penetration, and the atomization quality including the spatial and temporal distribution of droplet velocities and sizes. Accurate information on these parameters and droplet sizes in particular is an important factor in prediction of effectiveness of the nozzle operation. Droplet size is of special interest in many industrial applications including spray cooling, spray drying, spray washing, air conditioning, tablet coating, fire suppression, and agricultural spraying. Normally, the spray patterns come out with a range of droplet sizes. The smaller droplets exhibit more or less spherical shapes and are simply described by a diameter, whereas, the statistical methods are used to describe the overall droplet size distribution of the entire spray. One way to explain the droplet size distribution with single parameter is to use SMD. By definition, SMS is the diameter of a hypothetical droplet having volume to surface area ratio equal to that of entire spray distribution, and it is the best measure of the fineness of spray patterns [4].

The spray parameters discussed above are also used to forecast the development of spray patterns and droplets mixing rates and are well documented in the literature [2–4]. However, the induction of the vortex clouds and their effects on the development and quality of the spray structures have generally been overlooked especially in case of prespray heated liquid atomization [2]. Although, these vortex clouds play a key role in the rate of liquid evaporation, a nominal part of the research conducted so far has been devoted to the problem of the liquid jet evaporation, jet penetration accompanied by the semitorus like vortex clouds formation, and the interaction of the developing spray with the surrounding ambient air leading to the formation of the vortex clouds. These vortex clouds may have a significant influence on many important aspects of the liquid spray dynamics and should be studied in detail [5].

 Therefore, in this detailed paper, characterization of a laboratory scale intermittently forced hot water spraying system was carried out at temperature ranging from 20 to 100°C and load pressure ranging from 0.5 to 1.5 bar. The corresponding spray patterns were visualized using a high speed camera, whereas 1D PDA was used to study the SMD at different locations downstream of the nozzle exit. The generated data was analyzed in order to determine and optimize the important spray parameters like spray width, spray angle, spray tip penetration, Weber number, Reynolds number, droplet size, and so forth. The fine scale image analyses were also used to study the formation and collapse of the semitorus vortex clouds in the spray structures near the water boiling point. 

## 2. Materials and Methods

### 2.1. Development of Spraying System

**Figure 1:**
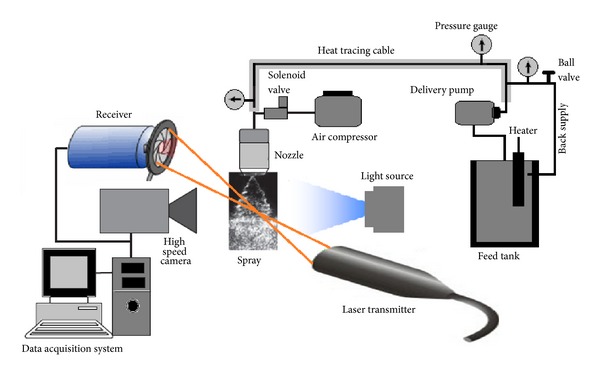
Schematic of the experimental setup used for generation and characterization of water spray.

Schematic of the experimental setup used for generation and characterization of water spray patterns as function of heating temperature and load pressure is shown in Figure 1. The in-house built spraying system was used to produce full cone spray patterns in a pulsed manner by means of a pulsed control system [2]. The system was furnished with three axi-symmetric full cone spray nozzles FC-2, FC-3, and FC-3.5 from RELAB having different orifice and free passage diameters as expressed in Table 1. These nozzles were equipped with a special X-shaped vane fixed at the nozzle inlet to impart the swirl and rotational speed to produce a full cone spray pattern. The spray pulse on-off duty cycle was controlled by a system composed of a PROVAL pneumatic double actuated solenoid valve and a programmable digital time relay (SIGMA, PTC-15). In these studies, the valve duty cycle was kept constant at 1 s. In order to maintain a desired temperature within the feed tank and spray feed line to spray point, the liquid immersion heater and heat tracing cable were used, and corresponding water temperature was monitored using thermostatic controllers. In this experiment, the service temperature was elevated from 20 to 100°C in order to lower the required load pressure, viscosity, and surface tension. In order to avoid the thermal losses, the heat tracing cable was insulated with ceramic tape. In addition to temperature, the service pressure of the system was being elevated in range of 0.5–1.5 bar. For this purpose, a liquid delivery pump capable of withstanding at high temperature values was used to serve the spray nozzle at its required spraying pressure. A stainless steel mesh strainer hampering any possible tiny debris from the liquid flow was also fixed at the feed tank outlet [2, 6]. The liquid pressure at three different localized points in main supply line was monitored using spring type pressure gauges.

### 2.2. Characterization Tools

In order to characterize the sprays of the tested liquid, the input parameters like heating temperature, load pressure, and liquid flow rate were varied in steps; the corresponding spray parameters including the axial spray tip penetration, jet breakup mechanism, droplet size distribution, nozzle discharge coefficient, spray cone angle, spray width, Weber number, Reynolds number, and vortex clouds formation were investigated using nonintrusive imaging and nonimaging tools like high speed camera and PDA. The visualization system used in this experiment was composed of a high speed digital camera and spray chamber side illumination arrangements. The transparent spray chamber was illuminated from all sides using 9 spotlights of 300 Watts each, and the spray jet movements were visualized using a Phantom v9.1 digital camera. This 14 bit-2 megapixels high speed camera suites well the larger field of view applications. It was capable of recording the 1,016 fps with a resolution of 1632 × 1200 pixels and simultaneously transferring the captured cine files to an image grabber. At a time, it can hold up to 24 GB of images [7]. Using this camera, the different spray parameters have been determined for each flow condition using frame by frame image processing method followed by manual verifications. The investigated parameters are the axial spray tip penetration, spray cone angle, spray width, nozzle discharge coefficient, Weber number, Reynolds number and vortex clouds. The discharge coefficient, Weber number and Reynolds number are given by [8]

(1) $$\begin{array}{c} C_{D}=\frac{v}{\sqrt{2\Delta p/\rho }}, \end{array}$$

(2) $$\begin{array}{c} We=\frac{\rho dv^{2}}{\sigma }, \end{array}$$

(3) $$\begin{array}{c} Re=\frac{\rho dv}{\mu }, \end{array}$$

where *v* is the mean flow velocity at nozzle exit, Δ*p* is the pressure difference, *d* is the nozzle exit diameter, *ρ* is the water density, *σ* is the surface tension, and *μ* is the dynamic viscosity.

 For droplet size measurement, a 1D PDA (from Dantec Dynamics) was used to measure the droplet diameters at different locations downstream of the nozzle exit. This technique permits the simultaneous measurement of droplet velocity and diameter. It is a nonintrusive technique and works on principle of measuring the light scattered by the particles. In the present case, PDA was composed of a CW Argon Ion Laser with *λ*1 = 514.5 nm, a transmitter, and a receiver. In order to minimize the contribution of reflected light, the receiving optics were placed at 30° to the forward scatter direction. The spray was scanned from 0 to 340 mm downstream of the nozzle exit with step size of 20 mm. The signals were processed by Burst Spectral Analyzer (BSA), and all data was transferred to a data acquisition system for further analysis.

## 3. Results and Discussion

### 3.1. Spray Jet Breakup Behavior

**Figure 2:**
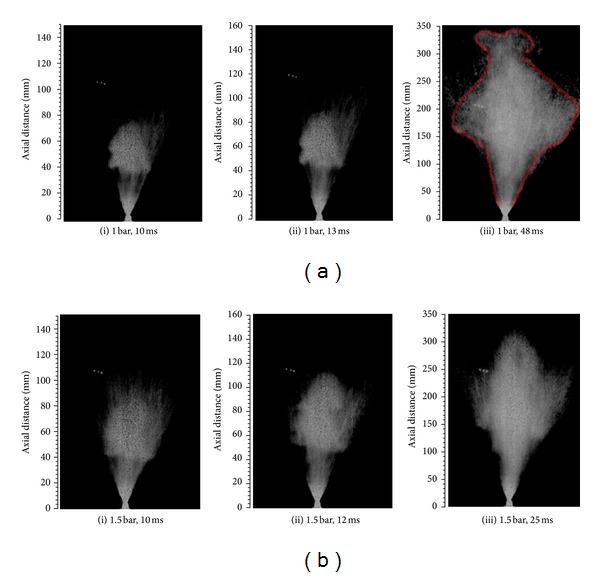
(a) Images of developing spray at 1 bar gauge pressure and 90°C service temperature. (b) Images of developing spray at 1.5 bar gauge pressure and 90°C service temperature.

**Figure 3:**
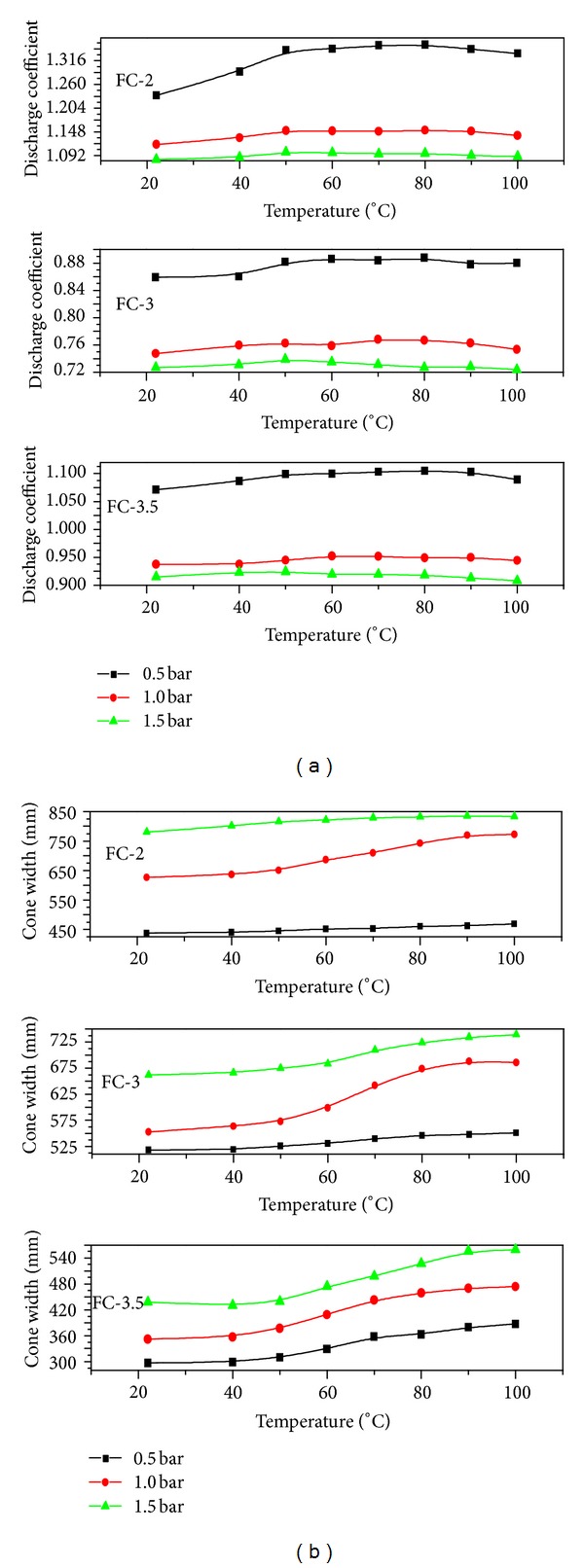
(a) Discharge coefficient as a function of temperature, (b) Spray width as a function of temperature.

 The photographic characterization of the water spray jet energized by axi-symmetric full cone nozzles was conducted by using a high speed camera. Some of the selected images grabbed from FC-2 nozzle at 1 and 1.5 bar load pressures and 90°C heating temperature are presented in Figures 2(a) and 2(b). In this study, three axi-symmetric full cone spray nozzles were investigated in the pressure range of 0.5–1.5 bar and temperature range of 20–100°C. Such nozzle works well only under fully developed liquid flow profiles. With higher load pressures, the instabilities occur in the water stream. These instabilities are caused by the local vorticities in the liquid flow. Therefore, the equation of the density dependent mass flow rate within the nozzles can be expressed as [9]

(4) $$\begin{array}{c} \dot{m}=C_{D}A\cdot \sqrt{2\cdot \rho \left(\Delta p\right)}, \end{array}$$

where **ρ** is the water density,* Q *is the volumetric flow rate, *C*_*D*_ is the discharge coefficient, *A *is the cross-sectional area of the orifice, and Δ*p* is the pressure difference. The derivation of above equation involves the nozzle orifice opening area, and the use of such small cross-sectional areas at the vena contracta is not the realistic approach. In addition, the nonnegligible fractional forces, turbulence, and viscosity effects may also have the adverse effect on the mass flow rates. Therefore, the term discharge coefficient was introduced in these investigations. The discharge coefficient is the ratio of the mass flow rate at the discharge end of the nozzle to that of an ideal nozzle. The discharge coefficient as a function of injection pressure and temperature is shown in Figure 3(a), where a decreasing trend was observed in the discharge coefficient with an injection pressure for all tested nozzles. With rise in injection temperature, initially it gave an incremental trend and then reached to a steady state above 50°C similar to mass flow rate and mean flow velocity. It was predicted that the FC-3 nozzle had the lowest discharge coefficient followed by FC-3.5 and FC-2. 

Normally, partial evaporation of the liquids in the system is stimulated by introduction of thermal energy below the liquid boiling point. The obtained vapor contents depend on the process parameters like, the degree of heating, pressure, and nozzle geometry. When the liquid is discharged into the surrounding environment with a phase inversion, the jet disintegration takes place, and the vapor phase inside the nozzle supports the disintegration process [10]. Therefore, in comparison with highly pressurized atomization, uniform spray patterns came out with a steadily increasing spray width as shown in Figure 3(b) and constant spherical droplet size distribution owing to the small droplet diameters. In these studies, an increment trend in spray width with temperature was more prominent at 1 bar pressure. This behavior strengthens the concept of liquid heating for improved atomization rather than subjecting very high load pressures. By doing so, the gas phase additives can be omitted due to availability of the vapor phase in spraying medium. By observing the mass flow rate and mean flow velocity as an integral parameter of the airless spray process, the occurrences inside the atomizer can also be investigated. 

**Figure 4:**
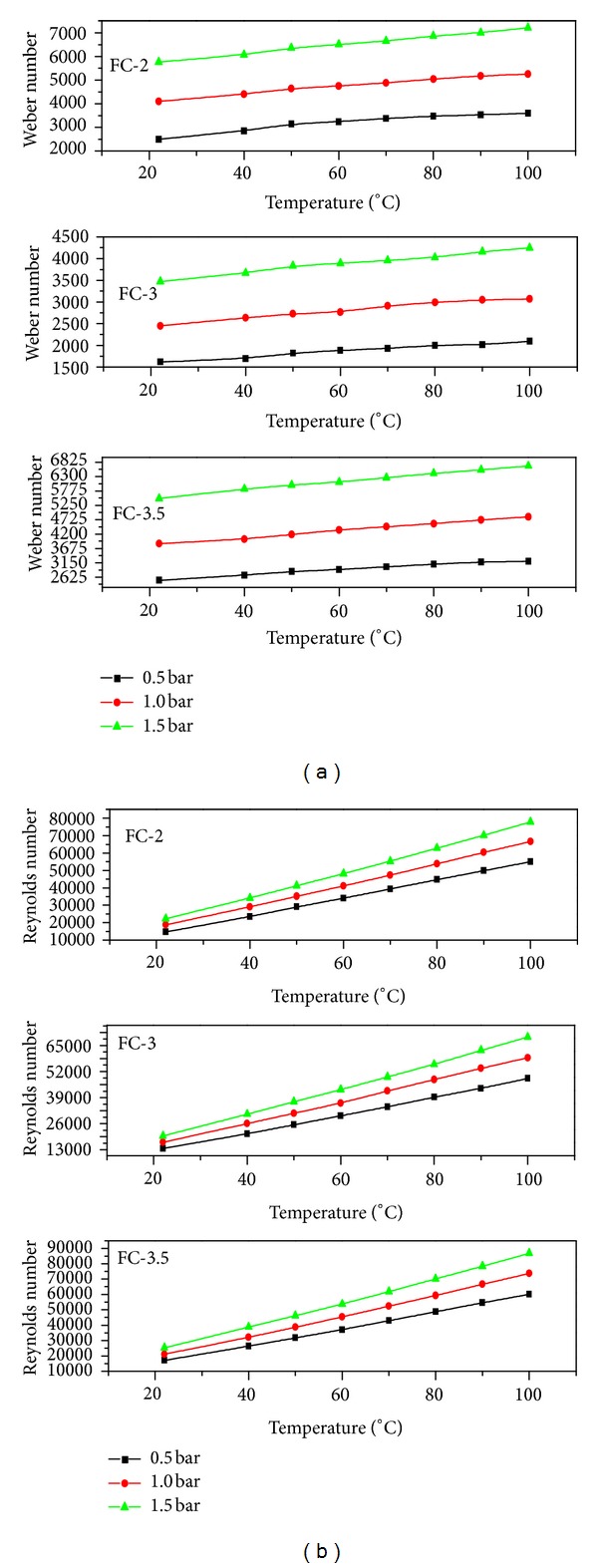
(a) Weber number as a function of temperature, (b) Reynolds number as a function of temperature.

From the images in Figure 2, the overall heat assisted atomization was regarded as efficient and pressure moderated. Apart from the parameters discussed so far, the other flow regimes can also be achieved by use of hot liquids as spraying media [11]. The most critical spray parameters involve the liquid flow within the atomizer and the interaction between the liquid jet and the ambient air. The liquid flow within the actuator and atomizers can be described by dimensionless quantities called Weber and Reynolds numbers. Figures 4(a) and 4(b) showed a monotonic increase both in Weber and Reynolds numbers which lead to an improved atomization and macroscopic spray cone length. The Reynolds number determines whether the liquid flow was dominated by inertial or viscous forces and hence either the flow was laminar or turbulent.

 The Reynolds number normally describes three mechanisms of liquid jet breakup [5]. Firstly, at low Reynolds numbers large uniform droplets are produced according to the Raleigh mechanism of jet breakup. Secondly, at intermediate Reynolds numbers, the breakup is achieved by jet oscillations with respect to the jet axis until the jet disintegrates into ligaments and then small droplet [12]. The second regime produces a wide range of droplet sizes. Finally, at high Reynolds numbers, the complete atomization of the jet is achieved within a short distance from the orifice as in case of FC-3.5 nozzle which had the highest Reynolds numbers among all the tested nozzles. 

The dependence of secondary atomization on relative velocities, liquid physical properties, and nozzle design is described by the Weber number. The Weber number is the ratio of the inertia of a fluid to its surface tension and hence is important in the process of droplet formation. Therefore the further droplet breakup and secondary atomization are expected to be strongly dependent on the Weber number [13]. In these investigations highest Weber number values were observed with FC-3.5 nozzle whiles the lowest with FC-3. 

**Figure 5:**
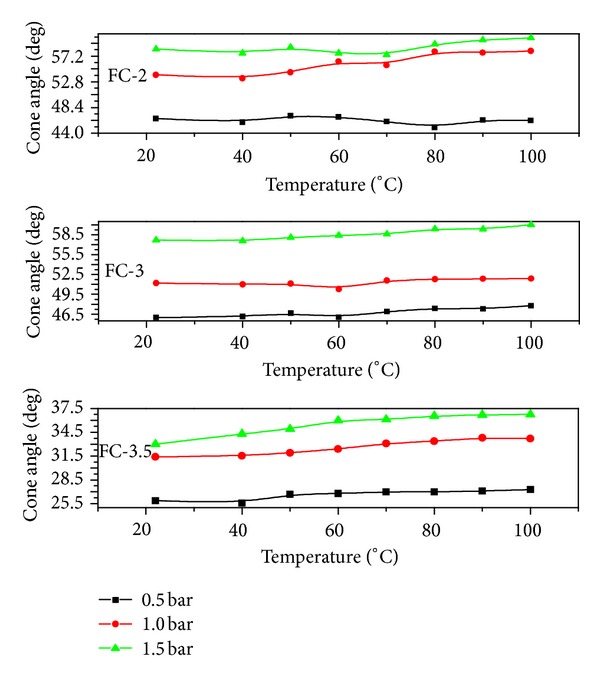
Spray cone angle as a function of temperature.

The spray cone angle was also measured against temperature using the mean value of 15 images as shown in Figure 5. The spray cone angle defines the spray boundary, and no variations were observed in the cone angle for 0.5 bar load pressure, while a slight incremental trend was seen at higher pressures of 1 and 1.5 bar. Since the spray cone angle investigations are usually carried out in a fully atomized mode, therefore, a decrease in the Weber number for a constant Reynolds number causes the spray cone angle to increase. This increase was considerably large at higher Weber numbers, while for low Weber numbers, the curves showed steady nature. So, it can be concluded that at high Weber number values, the spray cone angle becomes less dependent on the Weber number [14]. It was noticed during the spray visualization that from all three nozzles the main droplet streams divergence in the boundary rang from 27.5 to 61.2°, and if the droplet stream starts to shift its parameters out of this range, then it will be the sign of the malfunction or nozzle damage. Therefore, it is advisable that for each flow condition, the spray visualization and data analysis should be performed 5 times at least in order to assure the accuracy of the results obtained for the spray cone angle. In these studies, the maximum 7.6% error was noticed in spray cone angle while performing the error analysis.

### 3.2. Study of SMD of Droplets

**Figure 6:**
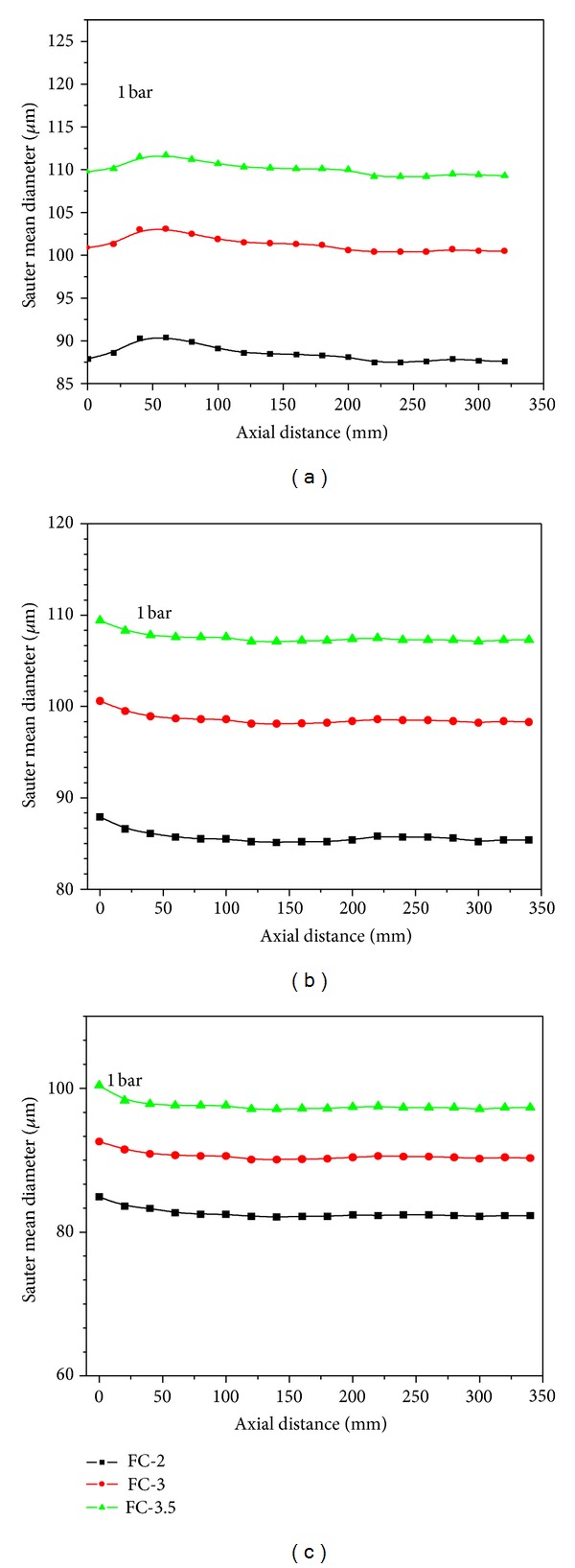
SMD as a function of axial distance from the nozzle tip for fixed temperature of (a) 20°C, (b) 60°C, (c) 90°C.

In this section, evolution of the droplet sizes from water spray patterns was provided. It was noticed that at low temperatures and pressures, the validation rates were also low due to an incomplete atomization and ligaments in the main stream. At higher temperature and pressure values, very dense spray patterns came out and obscured the signals. The axial profiles of SMD from all three nozzles are plotted in Figures 6(a), 6(b), and 6(c). The droplet size measurements were carried out at 18 axial stations downstream of the nozzle exit with step size of 20 mm.  Figure 6(a) reveals that at room temperature, SMD varies slightly at early injection stage and then reaches to almost constant values after moving 120 mm downstream. In this case, the smallest SMD values were achieved with FC-2 nozzle followed by the FC-3 and FC-3.5. It indicates that the orifice diameter plays key role in disintegration of liquid jet at room temperature. The smaller the orifice diameter is, the finer the spray pattern with small and spherical droplets will be. But the situation was changed when SMD was measured at high temperature values. Fully developed spray patterns with uniform droplet sizes were noticed as expressed in Figures 6(a) and 6(b), where SMD shows more likely monodispersed behavior. A clear decrease in droplet diameters was evident at higher temperature values regardless of axial location, load pressure, and orifice diameter. At fixed pressure of 1 bar, SMD values from all three nozzles were approaching each other significantly. The difference in SMD became less prominent at water heating near its boiling point temperature.

The water heating plays most dominant role in case of droplet sizes, no matter what was the driving pressure, nozzle diameter, and measuring position. Therefore, with increase of heating temperate, SMD was decreased and showed close approach for all nozzles regardless of the orifice diameter. It was also an indication of monodispersed nature of the droplet sizes. It is a valid conclusion in investigation and correlation of orifice sizes and SMD values at very high temperatures. These observations were also found consistent with those of Peter et al. [6], Brown and York [7], Bushnell and Gooderum [15] Park and Lee [16], and Gemci et al. [17]. In their studies, a decrease in mean diameter sizes was observed with increase of temperature at constant back pressures. The measurement of droplet sizes by Nagai et al. [18] at 250 mm downstream of the nozzle exit also confirmed a decline in SMD with rise of heating temperature.

### 3.3. Study of Vortex Clouds in Spray Patterns

At 90°C of water temperature, an abrupt change in jet velocity and penetration was noticed in 5–50 ms range of injection time. It predicts the droplets cloud formation during the atomization process as shown in Figure 2. These semitorus like clouds were located on the outer edge of the spray cone and were more prominent in case of FC-2 nozzle. Near the water boiling point temperature, the formation of such clouds might be natural but is still very complex phenomena to understand completely. Soon after formation, the vortex clouds moved, twisted, stretched, and interacted in a very complex way. Within the vortex core region, the jet flow was no longer irrotational and the angular velocity became nonzero roughly in the direction parallel to the vortex line. One can predict that it was due to the creation of pressure gradients in the ambient air which forced the jet flow to momentarily curve around the axis. In reality, the vortices are always composed of a core region that surrounds the axial line where the velocities of the spray droplets stop increasing and then decrease to zero as radius approaches to zero [17]. It was observed that the clouds formed at early injection stage vanish soon after creation, and the spray patterns start regaining uniform shapes. The droplets in these moving clouds are supposed to carry some mass, energy, and linear and angular momentums. The droplets rapidly lose their momentum and energy on account of the aerodynamic interactions among spray species. But in an ideal case, these quantities should never be dissipated, and the vortex clouds must persist forever [18]. 

Photographic study of the spray patterns also confirmed the presence of three phases in main flow at 90°C heating temperature. A developing phase with high jet velocity was noticed during early injection stage. In the 2nd phase, the spray droplets were decelerated by losing their momentum, and semitorus like structures were formed in the open air atmosphere. These clouds were translated and shattered as the jet collapsed towards the injector axis [19]. The 3rd phase was representing a quasisteady state where the mass flow rate was almost constant and the leading edge of the spray penetration was noticed to move linearly over time. At 1 bar pumping pressure, single vortex cloud was seen at early injection stage from where 2nd cloud was emerged after 30 ms of injection time. The 2nd vortex cloud was very prominent after 40 ms injection time and was lasted long compared with first cloud. On the other hand, multiple step vortex clouds were seen for 1.5 bar pumping pressure. In a later stage, these step clouds were changed into leaf like structures and then into fully developed spray patterns. The induced vortex clouds were collapsed and changed into a fully developed spray pattern after 100 ms of the injection time [20]. 

## 4. Conclusions

 In short, this paper reports the results of a spraying system tested with three axi-symmetric full cone spray nozzles at the temperature ranging from 20 to 90°C and the load pressure ranging from 0.5 to 1.5 bar. A high speed camera was used to photograph the images of generated spray patterns, whereas 1 D PDA was used to measure the SMD at different axial locations as a function of heating temperature at fixed pressure of 1 bar. The total scanned length downstream of the nozzle exit was 340 mm with step size of 20 mm. 

 The photographic study of the developing sprays confirmed that the injection of thermal energy into the system appears to be the most important spray boundary condition which strongly affects spray width, spray length, spray tip penetration, and other dimensionless parameters like Weber and Reynolds numbers. The fine scale image analysis also predicted the formation and decay of the semi-torus like vortex clouds in the spray structures near the water boiling point. For the smallest used orifice diameter (FC-2), these vortex clouds were more prominent at 1 and 1.5 bar water pumping pressure and at 90°C heating temperature. Single vortex cloud was noticed at early injection stage with 1 bar load pressure. After 30 ms from start of the injection, 2nd cloud was emerged from 1st cloud. At 1.5 bar service pressure, multiple step vortex clouds were observed in spray patterns which were changed into leaf like structures and then into fully developed spray patterns. 

 The PDA studies of the axial profiles confirmed that the droplet sizes decrease with increase in distance from the nozzle exit. It happened due to evaporation and breakup of large droplets into smaller one and spreading of the spray width downstream. The water heating played most dominant role in case of droplet sizes, no matter what was the driving pressure, nozzle diameter, and measuring position. As the heating temperate was increased, SMD was decreased and showed close approach regardless of the orifice diameter. It was also an indication of monodispersed nature of the droplet sizes. The measurement of SMD at room temperature leads to slightly bigger mean diameters for large orifice diameters. As the heating temperature increases, the effect of the orifice diameter on SMD becomes less prominent. These conclusions are valid in investigation and correlation of orifice size and SMD at very high temperatures. 

## Figures and Tables

**Table 1:** Specifications of full cone spray nozzles.

Sr. no.	Capacity code	Orifice diameter (mm)	Max. free passage diameter (mm)
1	FC-2	1.19	0.64
2	FC-3	1.59	1.02
3	FC-3.5	1.59	1.27
